# Temperature Thresholds Govern Microbial‐Mediated Dissolved Organic Carbon Dynamics in Coastal Ecosystems

**DOI:** 10.1002/advs.202511348

**Published:** 2025-10-15

**Authors:** Junfu Dong, Xiao Chen, Fangshuai Chen, Zhenzhen Hu, Qichao Tu, Gang Li, Chen He, Quan Shi, Qiang Zheng, Feng Chen, Tuo Shi, Nianzhi Jiao, Jihua Liu

**Affiliations:** ^1^ School of Life Sciences Shandong University Qingdao 266237 China; ^2^ Institute of Marine Science and Technology Shandong University Qingdao 266237 China; ^3^ Key Laboratory of Tropical Marine Bio‐Resources and Ecology South China Sea Institute of Oceanology Chinese Academy of Sciences Guangzhou 510301 China; ^4^ State Key Laboratory of Heavy Oil Processing China University of Petroleum Beijing 102249 China; ^5^ State Key Laboratory of Marine Environmental Science College of Ocean and Earth Sciences Institute of Marine Microbes and Ecospheres Xiamen University Xiamen 361005 China; ^6^ Innovation Research Center for Carbon Neutralization Fujian Key Laboratory of Marine Carbon Sequestration Xiamen University Xiamen 361005 China; ^7^ Environmental Science Center University of Maryland Baltimore MD 21202 USA

**Keywords:** dissolved organic matter, microbe‐DOM interaction, microbial community, ocean warming, temperature threshold

## Abstract

Temperature is a key factor influencing coastal carbon pools, yet the effects of warming on dissolved organic carbon (DOC) transformations and associated microbial processes remain poorly understood. Through bioassay experiments across a wide temperature gradient (7.6–35.9 °C), three critical thresholds (15.6, 24.6, and 29.9 °C) are identified that delineate distinct regimes of microbial DOC utilization with contrasting carbon fates. Below 15.6 °C, DOC characterized the most unique molecules, and their transformations are governed by bacteria whose relative abundance decreased with warming; hereafter warming‐resistant microbes dominated accompanied by DOC molecular signatures that changed till 24.6 °C. Limited substrate availability constrained microbial remineralization rates in these two stages. Once temperatures surpassed 24.6 °C, thermal‐favored microbes prevailed but taxa changed after 29.9 °C, DOC accumulated, and a larger fraction of recalcitrant DOC is retained, thereby enhancing carbon sequestration. Network analysis further revealed complex many‐one‐many resource‐consumer‐excretion linkages between bacteria and individual DOC molecules, underscoring the tangled nature of microbial DOC processing. The findings demonstrate that coastal DOC cycling responds to warming in a strongly non‐linear, threshold‐controlled manner, providing critical insights for predicting the behavior of coastal carbon sinks under ongoing climate change.

## Graphical abstract

Coastal dissolved organic carbon (DOC) represents one of the largest reduced carbon pools on Earth, and they are influenced by temperature. Across 7.6–35.9 °C ranges, the microbial‐mediated DOC dynamics is characterized by three temperature thresholds. Within varied temperature ranges, the dissolved organic matter molecular components and microbial community, as well as their interactions are different. These results demonstrate that the DOC cycling will be non‐linear in responding to global warming.

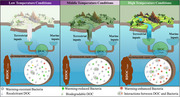

## 1 Introduction

Marine dissolved organic carbon (DOC) represents one of the largest reduced carbon pools on Earth.^[^ ^1^, ^2^ ^]^ A fraction of this DOC can be quickly remineralized to CO_2_ through microbial and photochemical processes,^[^ ^1^ ^]^ contributing to the vast dynamics of marine DOC.^[^ ^3^, ^4^ ^]^ Similar to most other biological processes, the transformation of marine DOC is temperature‐dependent.^[^ ^5^ ^]^ But these processes are unpredictable due to varied ambient conditions and disturbances from the climate change and anthropogenic activities, especially in coastal ecosystems.^[^ ^6^, ^7^ ^]^ The coastal ecosystem has a higher carbon sink potential facing the rise in atmospheric CO_2_, with a flux density (−0.68 ± 0.14 mol C m^−2^ year^−1^) 2.39 times higher than the open ocean.^[^ ^8^ ^]^ It is therefore essential to evaluate the impacts of temperature on coastal carbon cycles under different ambient conditions.^[^ ^9^, ^10^ ^]^

Molecular components of dissolved organic matter (DOM) are the key to deciphering the marine carbon cycles.^[^ ^2^, ^11^ ^]^ Some of the marine DOM molecules, e.g., the widespread carboxyl‐rich alicyclic molecules (CRAM) in the deep ocean,^[^ ^12^, ^13^ ^]^ exhibit high resistance to microbial degradation, known as refractory DOM (RDOM).^[^ ^11^ ^]^ By contrast, a substantial fraction of surface ocean DOM is readily consumed by heterotrophic bacteria as the main substrate and energy source,^[^ ^1^, ^14^, ^15^ ^]^ but becomes effectively non‐biodegradable when its concentration falls below the dilution threshold of ≈3 pm for an individual compound.^[^ ^2^, ^14^, ^16^ ^]^ Thus, the marine DOM is a complex mixture of truly recalcitrant molecules and thousands of labile species present at sub‐threshold concentrations.^[^ ^14^ ^]^ Resolving DOM composition at the molecular level is essential for deciphering marine carbon transformations.

The marine bacteria dominated the microbial processes of DOC transformations.^[^ ^3^, ^17^ ^]^ ≈50 Pg C from CO_2_ is fixed annually by marine phytoplankton into organic matter, nearly all of which is quickly mineralized back to CO_2_ by marine heterotrophs.^[^ ^2^, ^18^ ^]^ In the mineralized processes, organic matter shaped the consumer communities,^[^ ^19^, ^20^ ^]^ but were also influenced by the selective utilization and excretion of consumers.^[^ ^15^ ^]^ These marine consumers are sometimes much more abundant than their cyanobacterial counterparts.^[^ ^21^ ^]^ Warming not only enhanced the microbial respiration,^[^ ^22^ ^]^ metabolisms,^[^ ^6^ ^]^ and growth,^[^ ^23^, ^24^ ^]^ but simplified the habitats and restricted nutrient supply.^[^ ^25^, ^26^ ^]^ As all the biological processes and microbial communities are affected by temperature,^[^ ^5^, ^27^, ^28^, ^29^ ^]^ a non‐linear relationship is usually seen between marine microbes and DOM.^[^ ^19^ ^]^ It is therefore concluded that the microbial processes played vital roles in marine carbon transformations, but the microbe‐DOM interactions are highly complicated due to the warming disturbances.

Aoshan Bay, located in the Yellow Sea at the margin of the Western Pacific Ocean, is a semi‐enclosed coastal ecosystem characterized by strong seasonal temperature variations and complex terrestrial‐marine interactions.^[^ ^30^ ^]^ The bay experiences temperatures ranging from 0 to 30 °C, providing a natural temperature gradient representative of global temperate coastal zones. In addition, warming of this area exceeds that of the global average sea surface temperature (SST), and there were unprecedented marine heatwaves occurred in this bay, with maximum intensity and duration.^[^ ^31^ ^]^ Its ecological setting, with significant inputs of both terrestrial DOC via riverine discharge and marine DOC influenced by phytoplankton blooms,^[^ ^32^ ^]^ creates a dynamic environment ideal for investigating temperature‐driven microbial processes in coastal organic carbon transformation. The semi‐enclosed nature also facilitates controlled sampling and minimizes confounding open ocean influences. Thus, Aoshan Bay serves as a natural laboratory to observe microbial and biogeochemical responses across relevant temperature ranges and environmental conditions common to many temperate coastal systems worldwide.

In this study, we explored the dynamics of DOM composition, microbial community, and their interactions in response to warming under varying ambient temperature conditions at the Aoshan Bay, aiming to i) evaluate the influence of temperature on DOC dynamics and identify pattern in DOC utilization by microbes with warming under varying temperatures, ii) detect the changes in microbial community under different temperature, iii) characterize DOM molecular components, and further decipher microbe‐DOM interactions. This study is delving into how temperature merges as a critical factor influencing coastal DOC sequestration potential, and through its modulation of DOM‐microbe interactions.

## 2 Results

### 2.1 DOC Concentrations Vary with Different Incubation Temperatures

**Figure 1:**
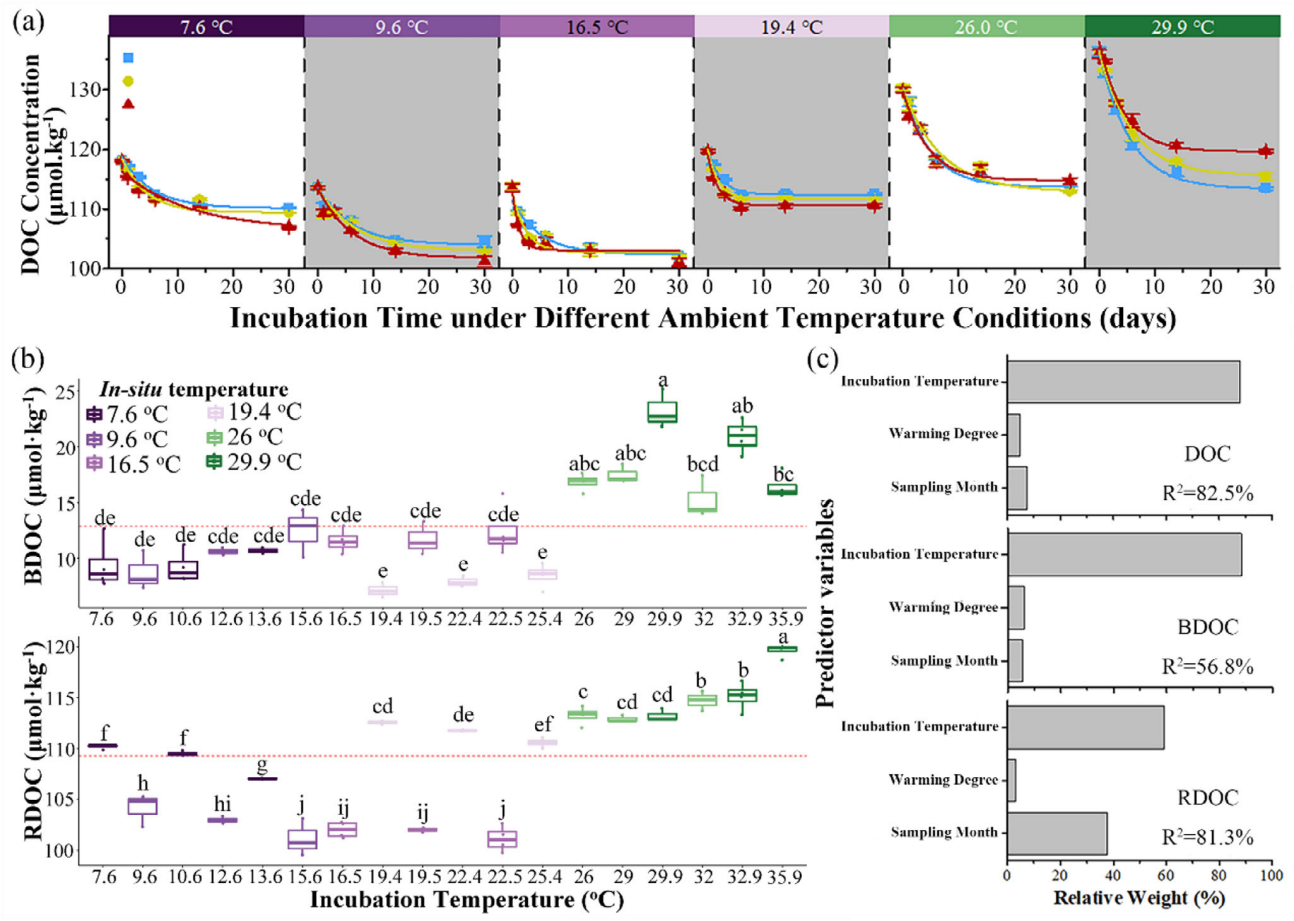
Transformations of DOC under different incubation temperatures. a) Changes in DOC concentrations with incubation time under different ambient temperatures. Error bars represent standard error (*n* = 4). The in situ ambient temperatures are indicated in the top panel. b) Variations in BDOC and RDOC concentrations under different incubation temperatures. Letters in lowercase indicate significant difference between temperatures. c) The relative weight (%) of important predictor variables for DOC, BDOC, and RDOC. R^2^ denotes the total percentage explained by predictor variables.

As the ambient temperature increased from 9.6 to 29.9 °C, the DOC concentrations showed increased trends, which was not the least under the lowest ambient temperature of 7.6 °C (**Figure** **1**a,b). The net loss and residual concentration in DOC were expressed by biodegradable DOC (BDOC) and refractory DOC (RDOC), respectively, by fitting the DOC decay kinetics to the first‐order model (see Experimental Section). The low BDOC concentrations were found at ambient temperatures of 19.4 and 7.6 °C, and warming effects on it were enhancing DOC degradation at 7.6, 9.6, and 19.4 °C, but enhancing DOC preservation at 16.5, 26.0, and 29.9°C (Figure 1a,b). Similarly, the low RDOC concentration was found at the ambient temperatures of 9.6 and 16.5 °C, and the warming effects were positive at 26.0 and 29.9 °C, but negative at 7.6, 9.6, 16.5, and 19.4 °C (Figure 1a,b). The DOC and RDOC concentrations were significantly correlated with the ambient temperatures (Figure S2, Supporting Information), but there were discrepancies in the warming effects on DOC concentrations at different ambient temperatures. The DOC transformations were primarily driven by the incubation temperature (rather than warming degrees or sampling months), which explains 87.8%, 88.3%, and 59.2% variance for DOC, BDOC, and RDOC, respectively (Figure 1c). In addition, compared to the initial DOC concentrations, the incubation temperature showed stronger correlations with BDOC concentrations (Figure S3, Supporting Information).

**Figure 2:**
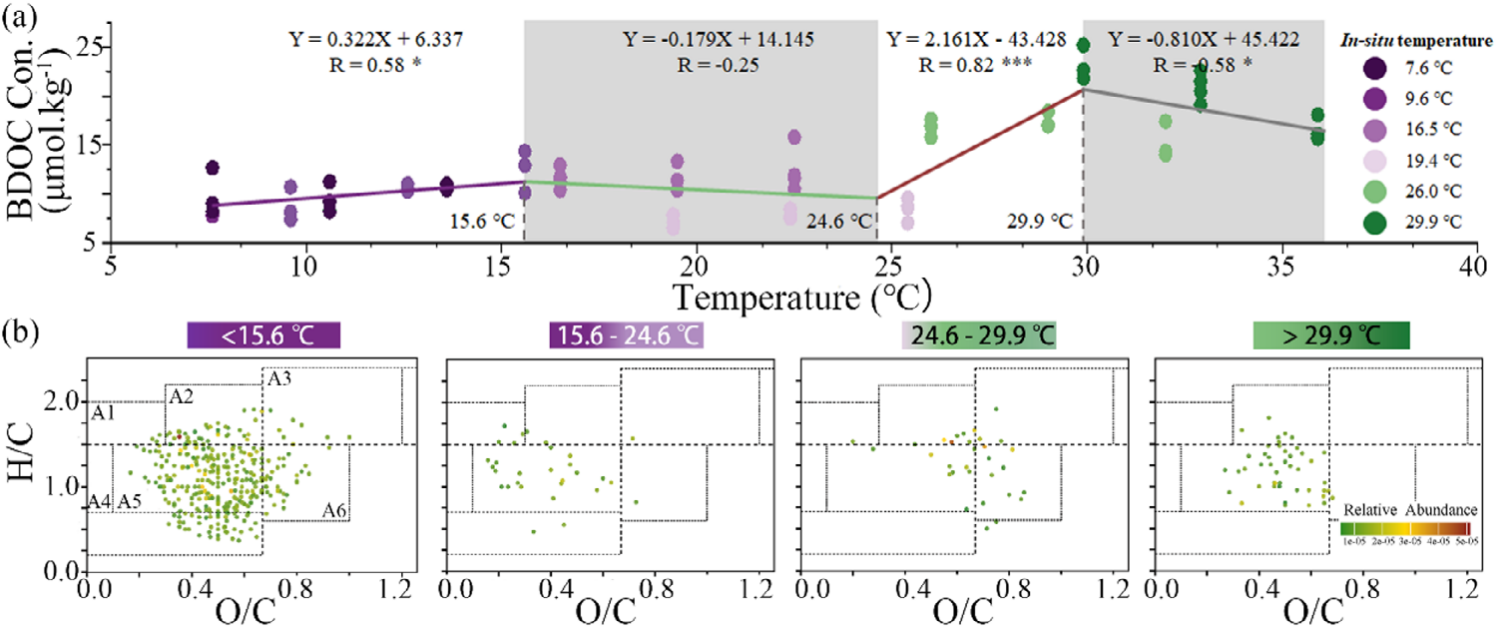
Segmented modelling for BDOC changes with increasing temperatures a) and unique DOM molecules within different temperature ranges b). Y represents the BDOC concentration (Con.) and X represents incubation temperatures. R indicates the correlation coefficient. ^*^, *p* < 0.05; ^**^, *p* < 0.01; ^***^, *p* < 0.001. The in situ ambient temperatures and relative abundance of DOM molecules are color coded. The DOM molecules in panel b represent the molecules before degradation experiments, those consumed by microbes, or those resistant to degradation. A1 to A7 represent lipids, aliphatic/proteins, carbohydrates, unsaturated hydrocarbons, lignins/CRAM‐like, tannins, and aromatic, respectively.

Based on the findings above, we further narrowed our focus on the DOC transformations with respect to incubation temperatures alone. The net loss in DOC fluctuated with increasing incubation temperature, which was best described by the three‐segmented model (71.38%) as compared to the linear and one‐segmented models (**Figure** **2**a; Figure S4 and Table S1, Supporting Information). As a result, four temperature ranges were obtained by three temperature thresholds (15.6, 24.6, and 29.9 °C) from 7.6 to 35.9 °C, and the loss rates of DOC were 0.322, −0.179, 2.161, and −0.810 µmol kg^−1^ °C^−1^ from low to high‐temperature ranges, respectively (Figure 2a).

### 2.2 Molecular Characteristics of DOM Differ Among Different Temperature Ranges

A total of 8481 DOM molecular formulas were detected with the Fourier transform ion cyclotron resonance mass spectrometry (FT‐ICR MS) technique, and their relative abundance was mostly (> 82%) between 0.01% and 1% (Table S2, Supporting Information), and the DOM composition was varied among sampling months (Figure S5, Supporting Information). > 65% formulas were assigned as lignins/CRAM‐like molecules, and the DOM components were different among the four temperature ranges. Specifically, the most abundant unique DOM molecules were detected when the incubation temperature was lower than 15.6 °C, most of which were lignins/CRAM‐like components (Figure 2b). Moreover, the molecular parameters (O/C)_w_, (DBE/C)_w_, and DBE_AI_/C_AI_ ratios were the lowest in value under the lowest incubation temperature ranges, but relatively high between 15.6 and 29.9 °C (Figure S6, Supporting Information). This trend was consistent with molecular proportion of RDOM but reversed for the H/C ratio (Figure S6, Supporting Information).

### 2.3 Temperatures Drive Changes in Bacterial Community Structure

**Figure 3:**
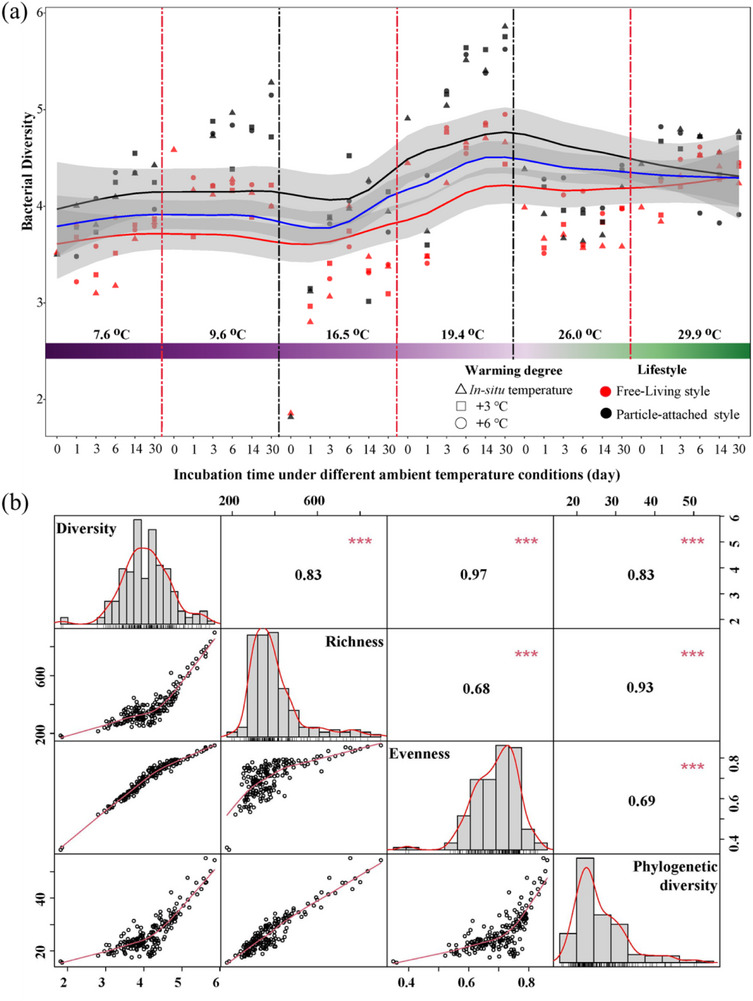
Changes in community diversity of free‐living (FL) and particle‐attached (PA) bacteria under different temperature conditions. a) Temporal dynamics of bacterial diversity. The fitted LOESS with 95% confidence intervals represents the mean of FL (red) and PA (black) diversity, as well as the mean of the total community (blue). The in situ ambient temperatures are indicated in the lower panel. b) Correlations between bacterial (Shannon) diversity, richness, evenness, and phylogenetic diversity. Numbers indicate the correlation coefficient (r). ^*^, *p* < 0.05; ^**^, *p* < 0.01; ^***^, *p* < 0.001.

The particle‐attached (PA) and free‐living (FL) bacterial diversity had the same trends with increasing incubation temperatures, but the diversity of PA was higher than FL (**Figure** **3**a). Changes in bacterial diversity were tightly correlated with evenness of the community, followed by its phylogenetic diversity and richness (Figure 3b). Proteobacteria, Bacteroidota, and Actinobacteriota were the most abundant phyla (up to 77.50–91.47%) that responded to increasing temperature (Figure S7, Supporting Information). However, only 2.91–36.70% of the responsive bacteria participated in the processes of DOM transformations, with the highest occurring at incubation temperatures ranging from 15.6 to 24.6 °C (Figure S8, Supporting Information). Based on the different responses to temperature increasing, microbes were categorized into warming‐resistant, reduced, and enhanced groups, and they primarily contributed to the DOM transformations at incubation temperatures ranging from 15.6 to 24.6 °C, 7.6 to 15.6 °C, and 29.9 to 35.9 °C, respectively (Figure S8, Supporting Information).

**Figure 4:**
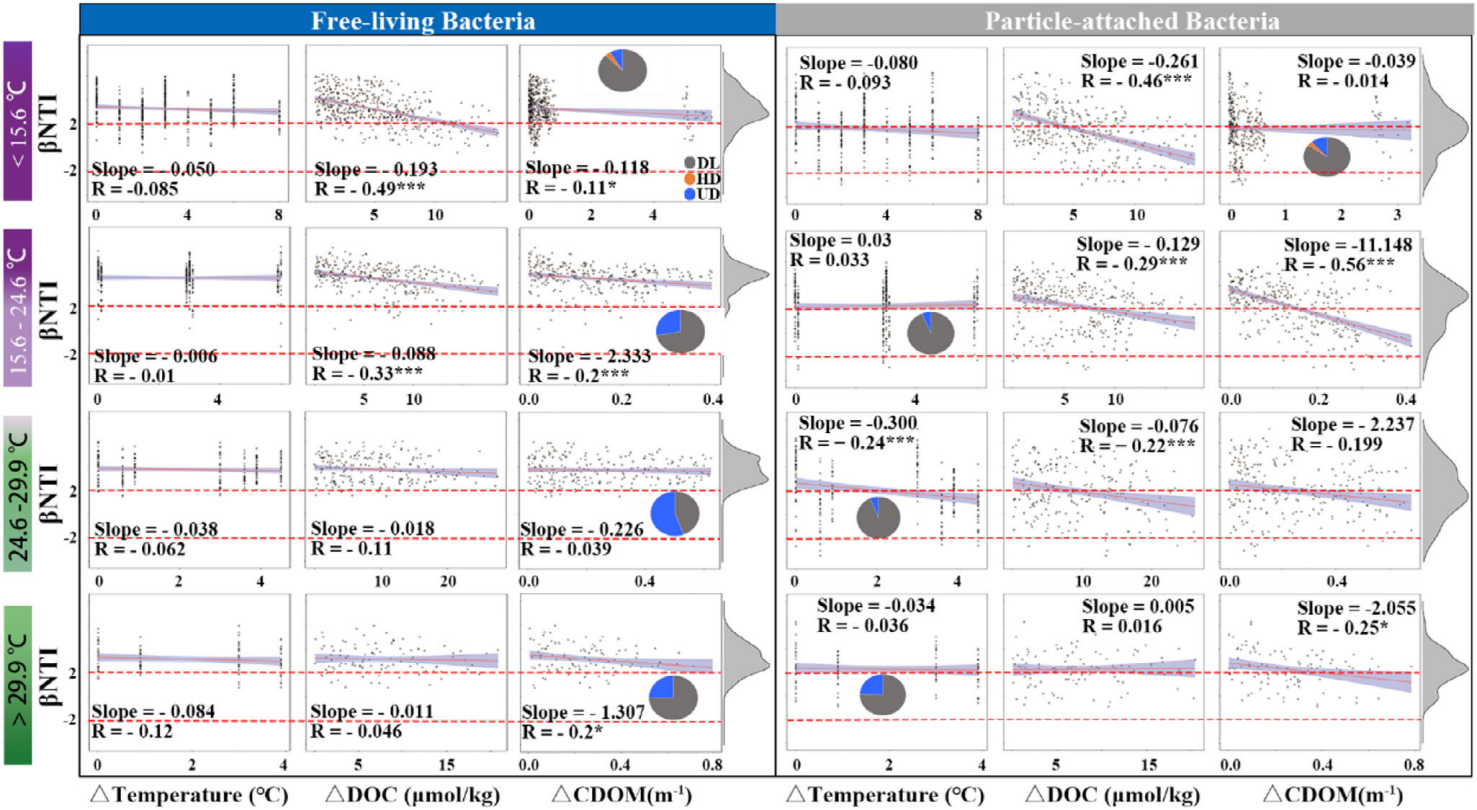
Relationships between βNTI and changes in incubation temperature, DOC, and CDOM for free‐living and particle‐attached bacterial communities within different temperature ranges. The βNTI density curves are shown on the right of each row. The pie charts show the relative percentage of stochastic processes for dispersal limitation (DL, gray color), homogenizing dispersal (HD, orange color), and undominated (UD, blue color). ^*^, *p* < 0.05; **, *p* < 0.01; ***, *p* < 0.001.

Variable selection processes govern the FL (83.67–96.10%) and PA (45.01–62.46%) bacterial communities, but the contribution of dispersal limitation was higher (29.52–45.45%) than that of FL (1.73–14.31%) (**Figure** **4**; Table S3, Supporting Information). As the vital environmental factors in this study, incubation temperature, DOC, and CDOM changed their roles in bacterial community assembly processes. Specifically, DOC mostly determined the FL bacterial community assembly at incubation temperatures ranging from 7.6 to 24.6 °C, but changed to CDOM when the incubation temperature increased beyond 29.9 °C (Figure 4; Table S3, Supporting Information). PA bacterial community assembly was shaped by CDOM in incubation temperature ranges of 15.6–24.6 and 29.9–35.9 °C, whereas that changed to DOC in incubation temperature ranges of 7.6–15.6 and 24.6–29.9 °C (Figure 4; Table S3, Supporting Information).

### 2.4 The Microbial Processes of DOM Transformations at Molecular Levels

**Figure 5:**
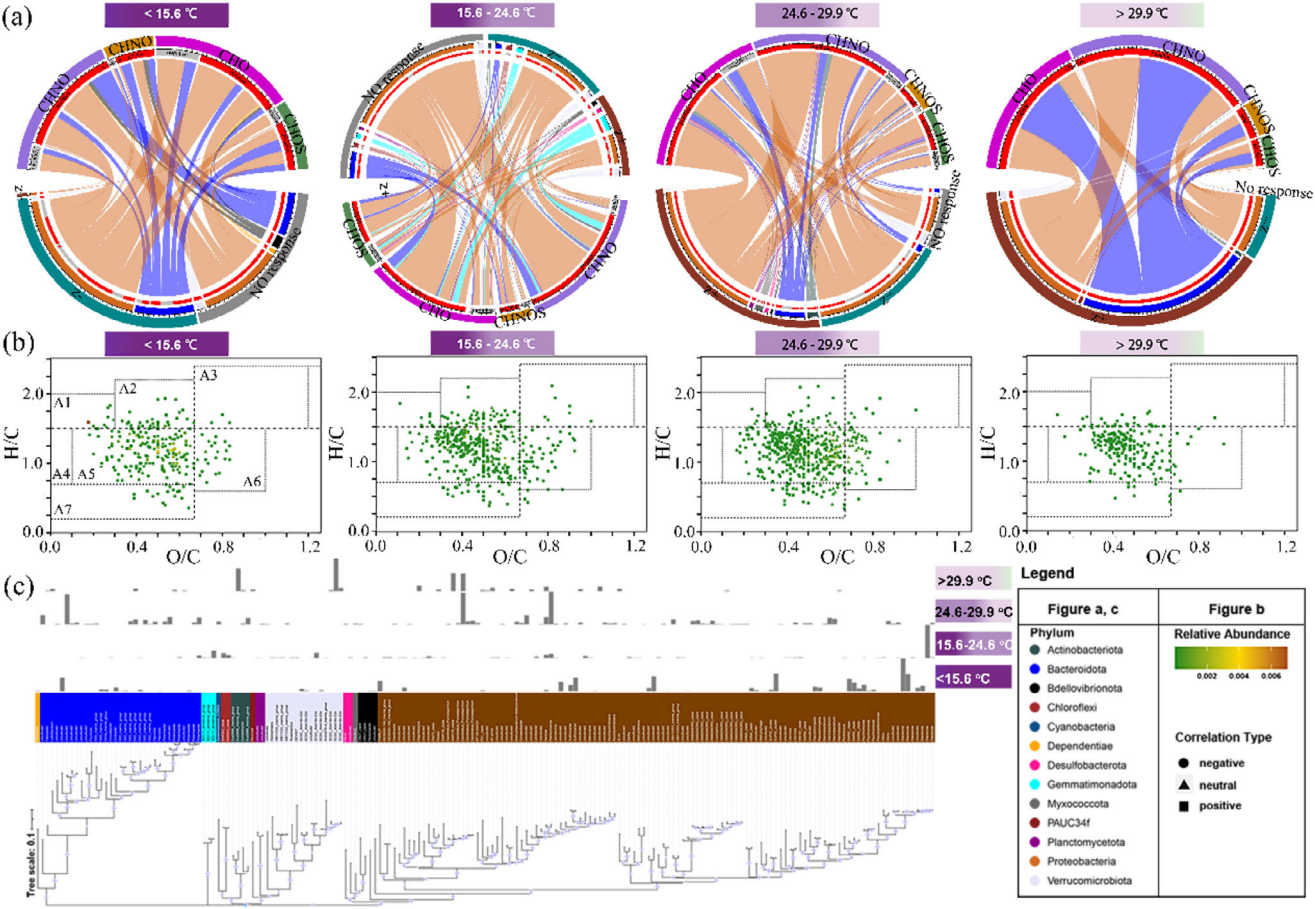
Interactions between bacteria and DOM molecules within different temperature ranges. a) Network of bacteria‐DOM interaction. The inner links depict the positive (red sectors) or negative (gray sectors) correlations between bacteria and DOM molecules; link width is proportional to the sum of absolute *r* values of Spearman correlations. Z‐ denotes warming‐reduced bacteria, Z+ denotes warming‐enhanced bacteria, and No response denotes warming‐resistant bacteria. Bacteria include both particle‐attached and free‐living styles. CHO, CHNO, CHNOS, and CHOS denote different components of DOM molecules. b) van Krevelen diagrams of the involved DOM molecules in the bacteria‐DOM interactions. A1 to A7 represent lipids, aliphatic/proteins, carbohydrates, unsaturated hydrocarbons, lignins/CRAM‐like, tannins, and aromatic, respectively. The DOM molecules in panels a and b represent the molecules before degradation experiments, those consumed by microbes, or those resistant to degradation. c) Phylogenetic analysis of the involved bacteria in the DOM transformations. Bacterial genera are labeled, and the phyla to which they belong are color‐coded. The relative abundance of bacteria is shown in the histograms corresponding to each temperature range.

The direct communications between bacteria and DOM occurred most frequently in the temperature range of 24.6–29.9 °C (1026 connections), followed by the ranges of 15.6–24.6 °C (933 connections), 29.9 to 35.9 °C (927 connections), and 7.6 to 15.6 °C (311 connections) (**Figure** **5**a; Figure S9, Supporting Information). In communications, the amounts of positive and negative correlations were all increased with temperature, but negative correlations were the least under the highest incubation temperature ranges (Figure S9, Supporting Information). These transformed DOM formulas were mainly assigned to lignins/CRAM‐like molecules, composed of CHO‐ and CHNO‐containing molecules (Figure 5a,b; Table S4, Supporting Information).

Proteobacteria (Alpha‐ and Gamma‐proteobacteria only) was the dominant phylum in the four temperature ranges, ranging from 1.4% to 24.8% (Figure S7, Supporting Information). Bacteroidota and Verrucomicrobiota also participated in the DOM transformations in the four temperature ranges, but their abundances were lower than Proteobacteria. The phyla Chloroflexi and Gemmatimonadota appeared at the incubation temperature ranges of 15.6–24.6 °C only with a 3.7–3.8% relative abundance, most of which were warming‐reduced/resistant groups; the direct communication results showed that these bacteria generated the CHO‐ and CHNO‐containing molecules after consuming the CHOS/CHNOS substrates (Figure 5a). Under the higher incubation temperature ranges, the warming‐enhanced bacterial groups contributed more to the DOM transformations. Specifically, Myxococcota of warming‐enhanced groups consumed CHO/CHNO with producing substances rich in N and S at incubation temperatures ranging from 24.6 to 29.9 °C, and PAUC34f of warming‐enhanced groups produced more CHNO/CHOS substances when the incubation temperature was above 29.9 °C (Figure 5; Table S4, Supporting Information). Overall, warming‐reduced and warming‐resistant bacteria played a critical role in DOM transformations when incubation temperature was lower than 24.6 °C, whereas warming‐enhanced bacteria were the main decomposers when incubation temperature was above 24.6 °C (Figure 5a, Table S4, Supporting Information).

## 3 Discussion

Microbes have been observed to convert DOC into CO_2_ or other various compounds,^[^ ^33^ ^]^ thereby effectively reducing the overall DOC pool. By using dark incubation conditions, we focused the DOC transformations specifically on microbial processes. To our surprise, the present study revealed that the net loss in DOC was primarily influenced by temperature, consistent with former findings,^[^ ^34^ ^]^ and structured by a three‐segmented model with warming: promoting DOC mineralization at 7.6–15.6 and 24.6–29.9 °C but reducing at 15.6–24.6 and 29.9–35.9 °C. The critical temperature thresholds that sharply alter microbial community composition and DOC dynamics challenge the common assumption of linear temperature effects in current coastal carbon models. From low to high‐temperature ranges, the temperature tipping points act as gatekeepers to allow warming‐reduced, warming‐resistant and warming‐enhanced microbes to metabolize DOC, respectively. Temperature structures the coastal DOC trends, which then shapes the microbial communities and microbial activities, affecting the DOC biotransformation by feedback and shifting the balance between carbon sequestration and release. Warming also results in varied DOC dynamics under different temperature ranges, which are positive or negative for carbon sequestration. These non‐linear responses were attributed to changes in the in situ DOC sources and the net remineralization rates,^[^ ^35^ ^]^ suggesting that existing carbon cycle predictions may misrepresent future coastal ecosystem behavior under warming scenarios, potentially underestimating or overestimating carbon sink strength.

As the main taxa, phylum of Bacteroidota had the highest abundance in all temperature ranges (Figure 5c; Figure S7, Supporting Information), suggesting a broad spectrum of DOC utilization and an independent lifestyle of this generalist.^[^ ^36^ ^]^ The second most abundant phylum was Verrucomicrobiota, and their occurrence was correlated to microalga diatom‐derived DOC.^[^ ^37^ ^]^ The other phyla of bacteria, Chloroflexi, Desulfobacterota, Gemmatimonadota, Bdellovibrionota, Proteobacteria, and Verrucomicrobiota, only presented under certain temperature ranges (Figure 5c). However, the bacterial community was mainly composed by rare species with cumulated abundance of 84.94%, while the total abundance of generalists only accounted for 15.6% (ranging from 1.86% to 54.08%), which was distinct to the natural ecosystems.^[^ ^38^, ^39^ ^]^ These alterations in microbial community composition were further proved their correlation with DOC rather than temperature changes in this study (Figure 4). It is, therefore, tempting to speculate that temperatures affected the DOC dynamics, hereafter exerting a selection pressure on the microbial community.^[^ ^40^ ^]^ Though the temperature was not the direct cause for microbial community alterations, it can potentially modify the microbial population by warming feedback loops with consequences for DOC transformations. We found that warming‐reduced and resistant bacterial groups played more vital roles in DOC transformations under lower temperature conditions, whereas warming‐enhanced bacterial groups dominated the community to be protagonists under higher temperature conditions (Figure 5c). Specifically, the warming‐resistant and reduced bacteria were the dominant groups consuming CHOS/CHNOS substrates when the temperature was below 24.6 °C, while the warming‐enhanced bacteria dominated the community in taking up more CHO/CHNO and producing more CHNO/CHOS substrates when the temperature was above 24.6 °C. These varied responses to warming consulting changes in bacterial community composition and their contributions to microbial processes. This chemical transformation presumably enhances CRAM's recalcitrance, with the incorporation of nitrogen into aromatic heterocycles introducing more robust bond architectures that resist downstream degradation.^[^ ^41^, ^42^ ^]^ However, because of the metabolic redundancy and concurrent,^[^ ^15^, ^43^ ^]^ we cannot precisely characterize the specific functions of these bacteria until now.

The greater cumulative abundance of rare species resulted in their contribution to DOC transformations reaching as high as 93.82%. These specialized bacteria exhibited diverse interactions with DOC. Previous research has suggested that Alphaproteobacteria excel at utilizing low‐molecular weight DOM, including glucose and amino acids, whereas Bacteroidia have a preference for high‐molecular weight DOM, such as chitin, N‐acetyl glucosamine, and protein.^[^ ^44^, ^45^, ^46^ ^]^ However, our study yielded no discernible disparity in the molecular weight of the DOM utilized by these two bacterial phyla. The molecular weight of their interacting DOM compounds shifted from a lower temperature range (453.39 Dalton for Alphaproteobacteria and 453.71 Dalton for Bacteroidia) to a higher temperature range (551.61 Dalton for Alphaproteobacteria and 556.21 Dalton for Bacteroidia). Furthermore, it was observed that the role of Gammaproteobacteria in the transformation of DOC was not significant, which contrasts with the previous findings;^[^ ^47^ ^]^ the functioning of Rhodothermia, Acidimicrobiia and Verrucomicrobiae in metabolizing CHO‐ and CHOS‐containing molecules were similar with recent findings,^[^ ^37^, ^48^ ^]^ but some of their functions were only present within specific temperature ranges. Overall, these findings highlight the important role of rare bacterial species in DOC transformations and the complex interactions between bacteria and organic matter in response to changes in temperature.

The interaction between bacteria and DOM can be classified as a consumer‐resource model, where bacteria serve as the consumers and DOM serves as the resource.^[^ ^17^ ^]^ This model can manifest in diverse patterns, encompassing one‐to‐one, one‐to‐many, many‐to‐one, and many‐to‐many relationships. Furthermore, the excretion patterns of bacteria can also affect the composition and concentration of the DOM pool. Some bacteria may excrete metabolic byproducts that other bacteria can use as a source of energy or nutrients, while others may excrete compounds that are toxic to other microbes. This can lead to complex resource‐consumer‐excretion patterns that can be difficult to untangle and understand. Our research discovered that most bacteria consumed (> 67%) and secreted (> 72%) more than one DOM molecule across all temperature conditions. However, most DOM molecules (57%) can only be consumed by specific bacterial species, confirming previous findings of specific one‐to‐one bacteria‐DOM relationships.^[^ ^45^, ^49^ ^]^ Moreover, these relationships were more prevalent when the temperature was below 15.6 °C in this study. Although many‐to‐many relationships have been observed in a pelagic marine system,^[^ ^15^ ^]^ they were not detected in our study due to differences in temperatures and spatiotemporal sampling resolutions. The correlation between specific one‐to‐one resource‐consumer and consumer‐excretion patterns underscores the critical impact of DOM on bacterial community assembly.

Apart from changes in the bacterial community and their interaction with DOM molecules, changes in the chemical composition of the DOM molecules as temperature increases also affect microbial metabolic potential and DOC dynamics.^[^ ^50^ ^]^ In comparison to high‐temperature ranges, DOM molecules were characterized by lower O/C and DBE/C, higher ratios in H/C and proportion of lignins/CRAM‐like molecules under low temperature ranges (Figure S6, Supporting Information). These observed structures imply that DOM was more easily broken down by microbes at lower temperatures. It may be conjectured that the lower BDOC content and bioavailability were a result of decreased concentrations of DOC and reduced metabolic rates of oceanic microorganisms. However, this study did not conclusively determine the relationship between these two factors. There was another result should be noted that there were the most abundant specific DOM molecules (332) when the incubation temperature was below 15.6 °C. But, out of these DOM compounds, only 16 molecules were found to directly interact with bacteria, and these interactions showed a positive correlation. This suggested that they were bacterial secretions and likely more resistant to degradation.^[^ ^17^ ^]^ The remaining 316 DOM molecules did not exhibit direct interactions with bacteria, possibly due to their lower normalized relative abundance (ranging from 0.128 to 0.0022 ‰) compared to the mean value of all samples (0.161 ‰), as well as their atom‐containing number pattern (more O: 9.647 ± 3.077 atom, less N: 0.763 ± 1.028 atom, less S: 0.274 ± 0.447 atom and less C: 19.780 ± 4.711 atom), which was similar to that of the deep‐ocean DOM.^[^ ^51^ ^]^ Additionally, 13 of these DOM molecules were categorized into the “island of stability,”^[^ ^52^ ^]^ indicating their higher resistance to degradation. Our findings have demonstrated that the reason for the reduced direct interactions between bacteria and DOM at lower temperatures was mainly due to the low concentrations of most DOM compounds at lower temperatures. At temperatures above 15.6 °C, the DOM molecules were found to be more recalcitrant, with higher O/C and aromaticity index, and a higher proportion of RDOM, but lower H/C (Figure S6, Supporting Information).^[^ ^52^ ^]^ However, despite the similarities in molecular properties across different temperature ranges, distinct DOM‐microbe interactions were observed. Specifically, the DOM molecules were more diverse, but showed fewer interactions with bacteria and lower concentration within the temperature range of 15.6–24.6 °C, which resulted in lower remineralization rates. This can be explained by the concentration‐driven uptake hypothesis.^[^ ^53^, ^54^ ^]^ Inversely, when the incubation temperatures were above 24.6 °C, DOM molecules contained higher levels of C and N but lower levels of O atoms. This higher reactivity, in conjunction with the increased DOM concentration, resulted in higher remineralization rates.

The temperate monsoon climate zone with high temperatures accompanied by high precipitations,^[^ ^55^ ^]^ and these climatic characteristics correspond to the growing season for nearshore plants, with more frequent rainfall events contributing to terrestrial DOC being directly transported to the coastal ecosystem.^[^ ^7^, ^56^ ^]^ The terrestrial inputs subsidized the DOC and nutrients for coastal ecosystems,^[^ ^57^, ^58^, ^59^ ^]^ improving marine DOC by stimulating phytoplankton growth.^[^ ^8^ ^]^ These two inputs contributed simultaneously to shape the seasonal dynamics of the coastal DOC pool in this study, and these dynamics were directly or indirectly affected by temperature, which was also proven by previous works.^[^ ^60^, ^61^ ^]^ But the coherence of temperature and precipitation and their combined impact on the seasonal variability of DOC make it challenging to separate their individual influences conclusively in a single year and limit our ability to deduct from correlation to causality. Correlation analysis showed that both incubation temperature and initial DOC show significant positive correlations with BDOC. However, the correlation with incubation temperature was stronger than that with initial DOC‐related (Figure S3, Supporting Information), highlighting the predominant influence of incubation temperature on DOC dynamics. Additionally, the mechanisms revealed in Aoshan Bay regarding microbial responses to warming and DOC dynamics remain capable of being generalized to other temperate coastal ecosystems with similar environmental drivers. Many coastal regions globally share features such as seasonal temperature fluctuations, mixed terrestrial and marine organic carbon sources, and microbial communities that govern carbon cycling. Therefore, identifying critical temperature thresholds and microbial community shifts here provides valuable insight into potential responses of coastal carbon pools under climate warming scenarios elsewhere. Moreover, the combined experimental approach of temperature‐controlled incubations and molecular bacterial‐DOC interaction analyses is fully replicable in other coastal contexts, facilitating regional and global comparative studies. We anticipate that the patterns observed in Aoshan Bay reflect fundamental biogeochemical principles that help inform carbon budget modeling and ecosystem management strategies across diverse coastal environments.

From a coastal management perspective, strategies aimed at maximizing coastal carbon sequestration often rely on predictable microbial responses to environmental factors. Our findings highlight that crossing such a temperature threshold can trigger abrupt shifts in microbial community and their processes, which could alter the effectiveness of these practices. As we have found, the microbial mineralization shows a bimodal pattern with temperature increasing from 7.6 to 35.9 °C, hinting at carbon sequestration with fluctuations. These indicate we can modify carbon sequestration by changing DOC concentrations under varied temperature ranges. While we do not prescribe specific adaptations, recognizing this tipping point encourages stakeholders to anticipate non‐linear ecosystem responses in policy and management, thereby enhancing the resilience of coastal carbon sinks amidst global climate change.

## 4 Conclusion

This study depicts the DOC fluctuations with increasing temperature by identifying critical thresholds where changes in temperature have a significant impact on DOC dynamics and its microbial processes. When the temperature was below 24.6 °C, the low DOC concentrations resulted in lower rates of microbial remineralization. Increasing temperature contributed to the increase in DOC concentration and metabolic rates of coastal microbes. The microbial processes of DOM transformations were mainly driven by different warming‐responsive bacteria groups in the four temperature ranges divided by the three temperature thresholds. Specially, warming reduced and resistant bacteria played more vital roles in DOC transformations under lower temperature conditions with lower magnitude of metabolic activity, whereas warming‐enhanced bacteria mediated the DOC transformation under the higher temperature range. However, the DOM molecular properties have changed between varied temperature ranges. In addition, the many‐one‐many patterns of the resource‐consumer‐excretion model entangle the microbe‐DOM interactions and DOC dynamics. Nevertheless, this finding is valuable in developing management strategies to preserve and enhance the ecological health of coastal ecosystems, including enhancing carbon sequestration.

## 5 Experimental Section

### Sampling and Dark Incubation Experiment

Aoshan Bay (36°20′N, 120°42′E, Figure S1a, Supporting Information), a semi‐closed coastal ecosystem at the Yellow Sea, was chosen as the permanent sampling site, where the historically recorded highest and lowest water temperatures were in August and January, respectively (https://www.ncei.noaa.gov/). The in situ SSTs were recorded using a Fluke 51‐II thermometer (Fluke Corp., Everett, WA, USA). Seawater was sampled from the surface in April, May, July, August, October, and December. Within 30 min after sampling, ≈200 L of seawater was transported in acid‐washed, polycarbonate (PC) carboys to the laboratory, where the seawater was immediately filtered through 20 µm pore‐size nylon mesh to remove large zooplankton and sediment particles, followed by filtration through 3 µm pore‐size 293 mm diameter cellulose ester films (Millipore Corp., USA) to remove bacteria‐feeding protozoa. The filtered seawater was then divided into nine 12 L transparent, acid‐washed, and pre‐combusted (450 °C, 4 h) glass bottles. The bottles were incubated in the dark for up to 30 days at three designated temperatures (in situ SSTs as ambient control, 3 °C higher, and 6 °C higher), ranging from 7.6 °C in December to 29.9 °C in August (Figure S1b, Supporting Information). Incubated seawater samples of 1000 mL each were collected on days 0, 1, 3, 6, 14, and 30, respectively. The seawater samples were filtered successively through 3, 0.8, and 0.2 µm pore‐size polycarbonate filters (Millipore Corp., USA). The fractions in the 0.2–0.8 µm size represent FL bacteria, and the fractions in the 0.82 µm size represent PA bacteria.^[^ ^62^ ^]^ These samples were used for the subsequent analyses.

April, May, July, and August were selected as sampling within different temperature ranges, with corresponding to 7.6–15.6, 15.6–24.6, 24.6–29.9, and 29.9–35.9 °C, respectively. Within each month, one in situ background sample was collected plus three temperature‐controlled incubations with one replicate. Sub‐samples were collected on days 1, 6, and 30, yielding ten samples per month (one background sample + three temperature levels × three timepoints × one replicate each) and a total of 40 high‐resolution samples across the temperature gradient.

For each sampling event, 500 mL seawater samples were filtered through 500 mL pre‐combusted (450 °C, 4 h) Whatman GF/F filters in an acid‐cleaned all‐glass filtration system under negative pressure. The GF/F filtrate were acidified to pH 2 using formic acid (Sigma–Aldrich, 98%) for DOM extraction. DOM was isolated via solid‐phase extraction (SPE) with a styrene‐divinylbenzene copolymer (PPL) sorbent.^[^ ^63^ ^]^ Specifically, the acidified filtrate (500 mL) was passed through the PPL cartridges (Agilent Bond Elut PPL, 500 mg, 6 mL) at a flow rate of ≈10 mL min^−1^ by gravity. Subsequently, cartridges were rinsed with three cartridge volumes of acidified Milli‐Q (pH 2, formic acid) to remove remaining salts and completely dried by flushing with ultrapure N_2_. Finally, the SPE‐DOM was eluted with 3 mL methanol and stored at −20 °C before FT‐ICR MS analyses.

### Calculation of Dynamics in DOC Concentrations

The concentrations of total DOC were measured with a TOC‐L CPH analyzer (Shimadzu, Japan) via high‐temperature catalytic combustion.^[^ ^64^ ^]^ BDOC and RDOC were calculated based on the first‐order model,^[^ ^65^, ^66^ ^]^ using the following equation:

(1) $$\mathrm{DOC}\;\left(t\right)=BDOC\;\times e^{-K_{DOC}\times t}+RDOC$$

where DOC(t) represents the concentration of DOC (µmol kg^−1^) at a specified timepoint *t* (day) during the course of the incubation experiment, whereas BDOC and RDOC represent the degraded and resistant pools (µmol kg^−1^), *K_DOC_* is the decay constant of DOC (day^−1^).

### Measurements of CDOM Concentrations

The concentration of chromophoric dissolved organic matter (CDOM) was measured using a UV‐2700 spectrophotometer (Shimadzu, Japan). Briefly, the UV absorbance of seawater was scanned at wavelengths ranging from 250 to 700 nm at 0.5 nm intervals using a 5 cm path length of the quartz vials, with ultrapure Milli‐Q water used as the blank. After subtracting the mean of absorption between 680 and 700  nm, the absorbance values were converted to Napierian absorption coefficients using the following equation:

(2) $$a_{\lambda }=2.303A_{\lambda }/L$$

where a_λ_ is the Napierian absorption coefficient (m^−1^), A_λ_ is the absorbance measured by the spectrophotometer, and L is the path length (m). The concentration of CDOM was derived from the UV absorption coefficient at 254 nm (a_254_), resulting primarily from macromolecules such as humic substances, colloids, carbohydrates, proteins, etc.^[^ ^67^ ^]^

### Characterization of DOM Molecular Components

The molecular formulas of DOM were analyzed using a 9.4 Tesla Apex‐ultra FT‐ICR MS (Bruker Daltonics, Billerica, MA, USA) coupled with negative‐ion mode electrospray ionization (ESI) according to Shi, et al.^[^ ^68^ ^]^ and Zhao, et al.^[^ ^44^ ^]^ More details about the FT‐ICR MS method can be found in the Supporting Information. Based on the DOC dynamics, we determined three temperature thresholds (15.6, 24.6, and 29.9 °C), which correspond to the monthly range of April (7.6–15.6 °C), May (15.6–24.6 °C), July (24.6–29.9 °C) and August (> 29.9 °C), respectively. This setting eliminates the influence of varied DOM composition and microbial community structures, enhancing the reliability of the conclusion of this study. As a results, the average of integrated data for one temperature range only encompasses the results of DOM composition collected from the same ambient conditions. ^13^C‐containing molecules were excluded when characterizing the molecular formulas.^[^ ^69^ ^]^ The frequency in occurrence for a DOM molecule at a specific temperature range was calculated based on a matrix of presence (1) or absence (0). DOM molecules were considered unique if their occurrence frequency was >50% under one temperature range but < 50% under the other temperature ranges. Molecular formulas were assigned to DOM according to the classification proposed by Sleighter, et al.^[^ ^70^ ^]^ and Koch and Dittmar ^[^ ^71^ ^]^ (see details in the Supporting Information). The molecules with a H/C<1.5 were categorized as RDOM.^[^ ^72^ ^]^ The DOM molecules were visualized with H/C against O/C by van Krevelen diagrams.^[^ ^44^ ^]^

### Assessment of Bacterial Community Structure

Bacterial DNA was extracted from 500 mL seawater using the phenol‐chloroform‐based DNA extraction method.^[^ ^62^, ^73^ ^]^ The V3‐V4 region of 16S rRNA gene was amplified with primers 338F/806R.^[^ ^74^ ^]^ More details for PCR cycling conditions, amplicon library construction and next‐generation sequencing with the Illumina NovaSeq platform are available in the Supporting Information. After quality filtering, the sequence reads were processed with QIIME 2 (version 2019.1).^[^ ^75^ ^]^ The low‐quality sequences and chimeras were discarded to generate amplicon sequence variants (ASVs) using DADA2.^[^ ^76^ ^]^ ASVs were classified based on the SILVA 16S rRNA database (v138) with the Naive Bayes consensus taxonomy implemented in QIIME 2.^[^ ^77^ ^]^ The sequence data was deposited to the National Genomics Data Center (NGDC) database under the accession number CRA004479.

The alpha diversity of the bacterial community was calculated using the package Vegan (v2.5.7). Phylogenetic clustering for PA and FL bacterial communities was evaluated using the R package picante (v1.8.2). The bacterial community assembly processes were evaluated using beta nearest taxon index (βNTI) and Bray‐Curtis‐based Raup‐Crick (RC_bray_).^[^ ^78^, ^79^ ^]^ Mantel's tests were performed to explore the determining factor of community assembly.^[^ ^80^ ^]^

Using z scores normalized based on the mean and standard deviation of permuted indicator values, the bacterial ASVs were classified into warming‐enhanced (z+), warming‐reduced (z‐), and warming‐resistant groups through the threshold indicator taxa analysis (TITAN) (v2.4.1).^[^ ^81^ ^]^ The method designed to detect taxonomic change points along environmental gradients via permutation‐based approaches, providing statistical confidence for each breakpoint detected.

The co‐occurrence networks between the warming‐responsive bacteria and DOM molecules in different temperature ranges were analyzed with the R packages Hmisc (v4.5.0) and igraph (v1.2.6) based on Spearman's correlations (*r*).^[^ ^82^ ^]^ To reduce the network complexity and improve the precision,^[^ ^83^, ^84^, ^85^ ^]^ only bacterial ASVs with a relative abundance >0.01% and present in >20% of the samples were analyzed. The significance of correlations was assessed using a threshold of *r*>0.9 and “BH” method‐adjusted *P* < 0.05. According to resource‐consumer relationships, the negative network correlations indicate decomposition of existing DOM molecules, while the positive correlations indicate generation of new DOM molecules.^[^ ^86^ ^]^ The co‐occurrence network of bacteria‐DOM interactions was created using the R package circlize (v0.4.13), and further visualized in Cytoscape (v3.8.2). Additionally, phylogenetic relationships among bacterial ASVs were inferred using the Qiime2 plugin “q2‐fragment‐insertion,” aligned to the Greengenes 13.8 reference database.^[^ ^87^ ^]^ The resulting phylogenetic tree was visualized online using Interactive Tree Of Life (iTOL, https://itol.embl.de/).^[^ ^88^ ^]^

### Statistical Analysis

All analyses were performed in R v4.1.0 (www.r‐project.org). The DOC concentrations and DOM molecular formulas at different incubation temperatures were compared using one‐way analysis of variance (ANOVA), coupled with the least significant difference (LSD) post hoc tests. All data were pre‐processed by normality and homogeneity tests. These experiments were designed based on three factors: sampling months, ambient in situ temperatures, and degrees of warming. Multicollinearity diagnostics were performed on the three predictor variables, and their relative importance with respect to the DOC concentrations were accessed based on their proportionate contribution to R^2^.^[^ ^89^ ^]^ The generalized additive model (GAM) was used to determine the number of temperature thresholds for BDOC concentrations, and the tipping points were then used to explore the explicit coefficients of the threshold models. The goodness of fit between different models were analyzed using the package segmented (v1.3.4). All data was presented by mean± SE.

## Conflict of Interest

The authors declare no conflict of interest.

## Author Contributions

J.L. and N.J. conceived and designed the experiment. X.C., F.C., and Z.H. carried out the experiments. J.D., J.L., and T.S. drafted the manuscript. All authors contributed to improving the interpretation of the data and approved the final version of the manuscript.

## Supporting information



Supporting Information

## Data Availability

The data that support the findings of this study are available from the corresponding author upon reasonable request.
