# Supplementary material for: Temperature Thresholds Govern Microbial‐Mediated Dissolved Organic Carbon Dynamics in Coastal Ecosystems
Source: Adv Sci (Weinh). 2025 Oct 15;13(2):e11348. doi: 10.1002/advs.202511348 (PMC12786288; doi:10.1002/advs.202511348)
Supplement: Supplementary file 1 — Supporting Information [file ADVS-13-e11348-s001.docx]

**Supporting Information**

**Temperature Thresholds Govern Microbial-Mediated Dissolved Organic Carbon Dynamics in Coastal Ecosystems**

This file includes:

1. Supporting Materials and Methods
2. Supporting Figures S1−S10
3. Supporting Tables S1−S5

**Supporting Materials and Methods**

**FT-ICR MS analysis**

The sample solution was infused into the ionization source at 250 μL/h. The mass limits were set between 200 and 800 Da for the m/z ranges, and all peaks with >4 for the signal-to-noise ratio were used to characterize the molecular formula [1]. These DOM formulas were divided into seven component classes (lipids, aliphatic/proteins, lignins/carboxylic rich alicyclic molecules (CRAM)-like structures, carbohydrates, unsaturated hydrocarbons, aromatic structures, and tannins) according to previously publications [2, 3].

For molecular formula assignments, we used the following equations according to Sleighter RL et al. [4] and Koch BP and Dittmar T [5]:

$${(\frac{O}{C})}_{w}= \sum(\frac{O}{C_{n}}\times M_{n})$$

$${(\frac{H}{C})}_{w}= \sum(\frac{H}{C_{n}}\times M_{n})$$

$$DBE=0.5\times(2\#C+\#N+\#P-\#H+2)$$

$${(DBE)}_{w}=\sum({DBE}_{n}\times M_{n})$$

$${(DBE/C)}_{w}=\sum({DBE/C}_{n}\times M_{n})$$

$${(DBE/O)}_{w}=\sum({DBE/O}_{n}\times M_{n})$$

$${(C\#)}_{w}=\sum({\#C}_{n}\times M_{n})$$

$$\mathrm{DBE}_{AI}/C_{AI}=\frac{1+\#C-\#O-\#S-0.5\#H}{\#C-\#O-\#S-\#N-\#P}$$

where ‘w’ represents the magnitude-weighted calculation, ‘n’ denotes that the calculation of parameter for specified formula, # indicates the number of the specified atoms in the formula, and ‘M’ represents the relative magnitude of each peak.

We classified all formulas of DOM into six categories based on their relative abundance following the classified thresholds [6, 7]. Specifically, 0.1 and 0.01% were set as the cutoff level of relative abundance. Always abundant formulas (AAF) represent the formulas having a relative abundance of ≥1% in all samples; conditionally abundant formulas (CAF) represents the formulas that had a relative abundance of ≥0.01% in all samples and ≥1% in some samples; always rare formulas (ARF) represent the formulas having a relative abundance <0.01% in all samples; conditionally rare formulas (CRF) represent the formulas having a relative abundance <0.01% in some samples but never ≥1% in any samples; moderate abundant formulas (MAF) represent the formulas having a relative abundance between 0.01% and 1% in all samples; conditionally rare and abundant formulas (CRAF) represent the formulas having a relative abundance ranging from rare (< 0.01%) to abundant (≥1%).

**16S rRNA gene amplicon sequencing**

The DNA was quantified and qualified using a Micro Spectrophotometer (Drawell, USA) and stored at –80 ℃ before use. The V3-V4 region of the bacterial 16S rRNA gene was amplified using forward primer 338F (5’-ACTCCTACGGGAGGCAGCA-3’) and reverse primer 806R (5’-GGACTACNNGGGTATCTAAT-3’), coupled with a unique 7-bp barcode sequence before the 5′ for multiplex sequencing. Polymerase chain reaction (PCR) was conducted in a 50 μL reaction with 25 μL 2× Premix Taq, 1 μL of each primer (10 μM), 20 μL nuclease-free water, and 3 μL template DNA (20 μg/μL). The reaction conditions of PCR were initial denaturation at 94 °C for 5 min, followed by 30 cycles with 30 s denaturation at 94 °C, 30 s annealing at 52 °C, and 30 s elongation at 72 °C, and lastly followed by 10 min at 72 °C. The purified sequences were used to generate the library using the NEB Next^®^ Ultra^TM^ DNA Library Prep Kit for Illumina^®^ (NEB, USA) according to the manufacturer's recommended protocol. The library was sequenced by the Illumina NovaSeq platform at Guangzhou Magigen Biotechnology (Guangzhou, China).

**Supporting Figures**


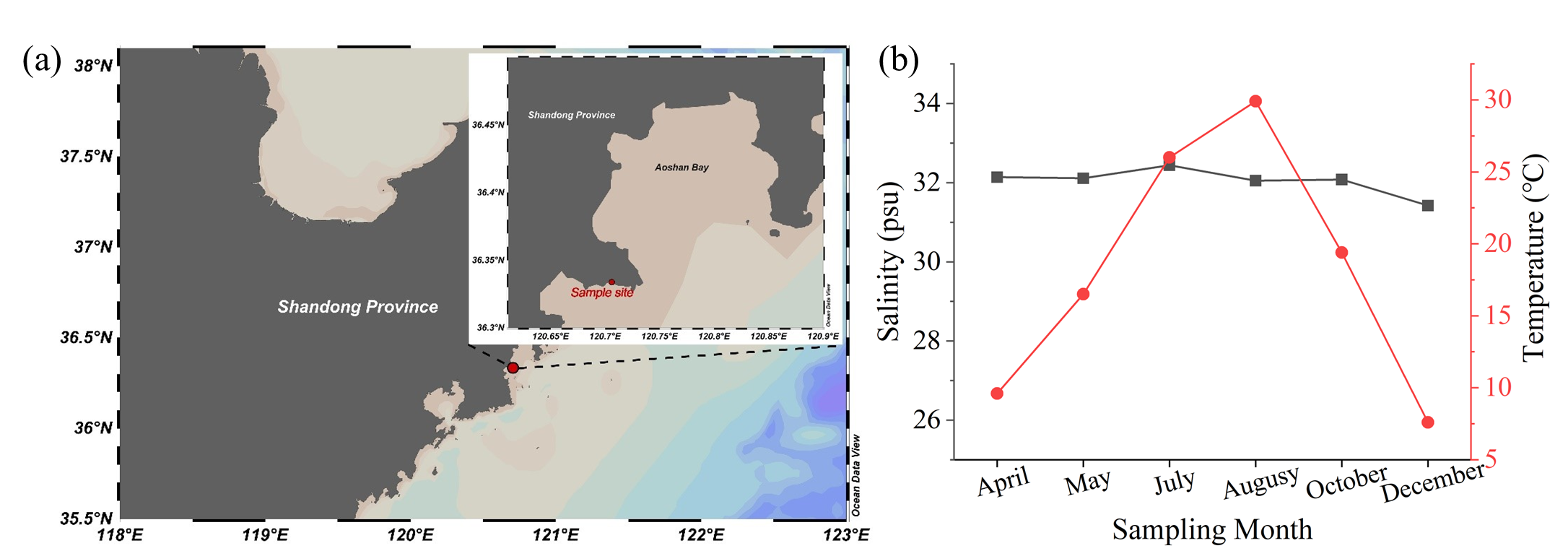


**Figure S1. The sampling location (a) and the *in-situ* ambient temperatures (b).**


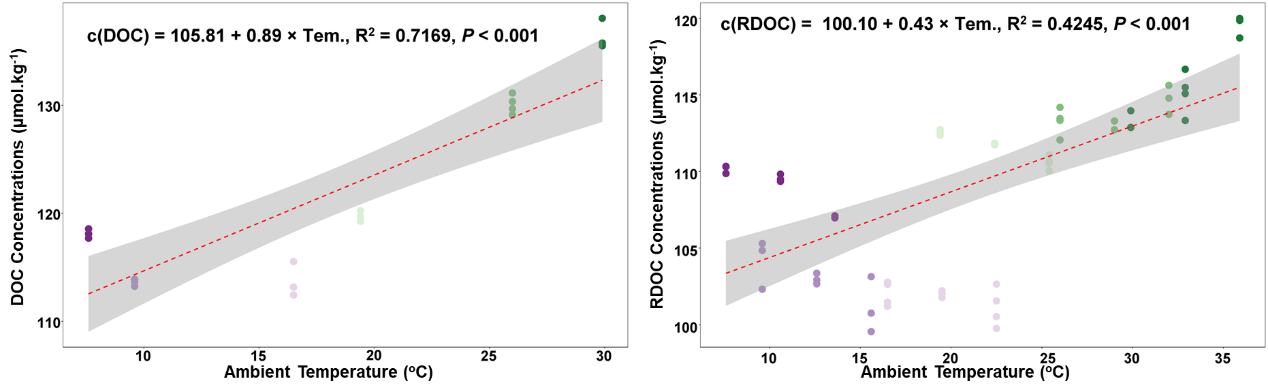


**Figure S2. Correlations between ambient temperatures (Tem.) and DOC or RDOC concentrations.**
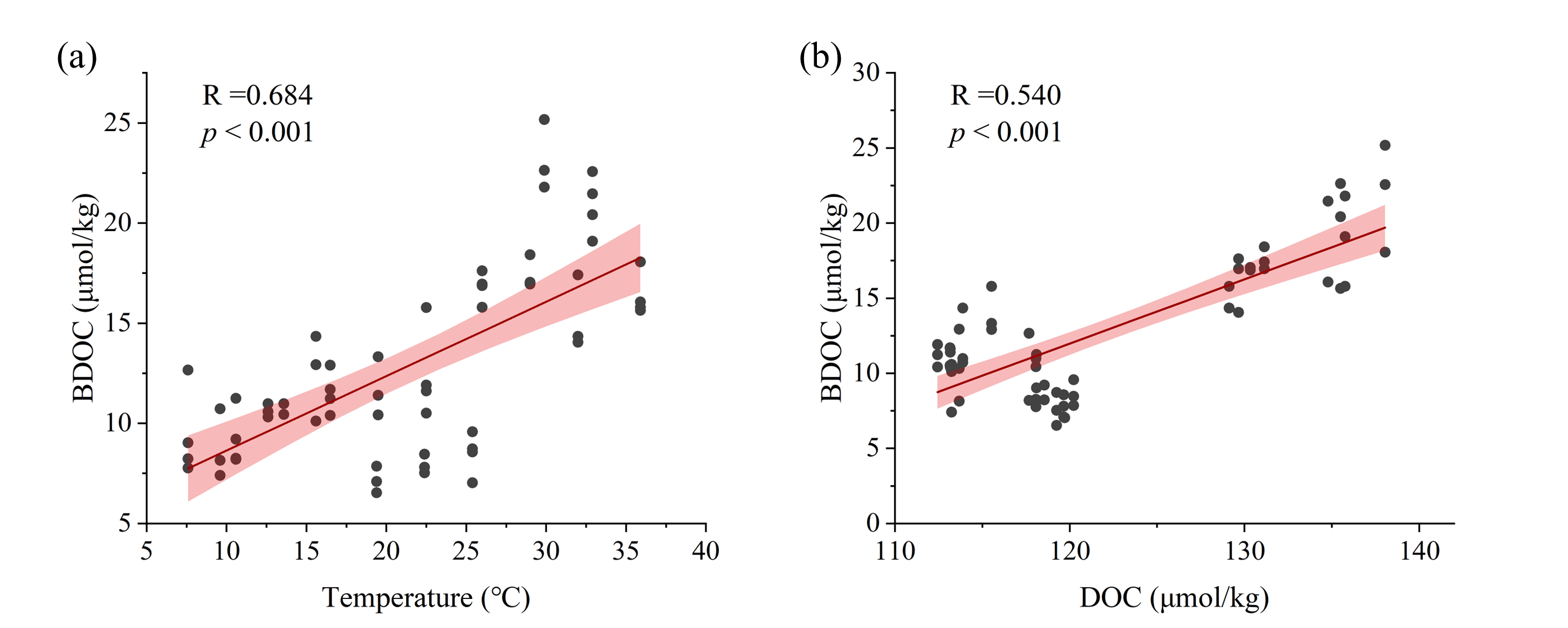


**Figure S3. Spearman’s correlations of biodegradable DOC (BDOC) with incubation temperature (a) and with initial DOC (b).**


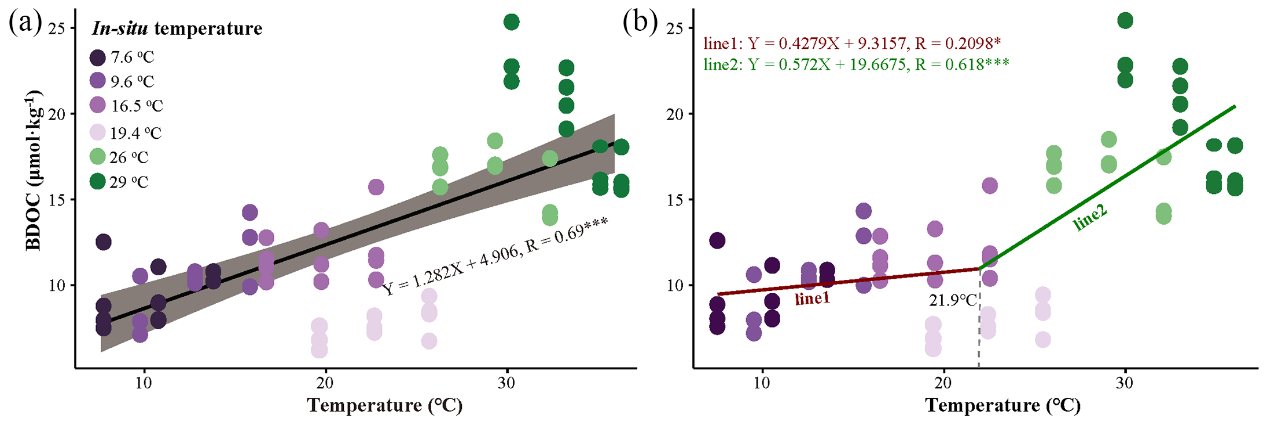


**Figure S4. The linear (a) and one-segmented (b) models for BDOC changes with increasing temperature.** Y represents the BDOC concentrations, X represents incubation temperatures, R indicates the correlation coefficients. *, *p* < 0.05; **, *p* < 0.01; ***, *p* < 0.001.


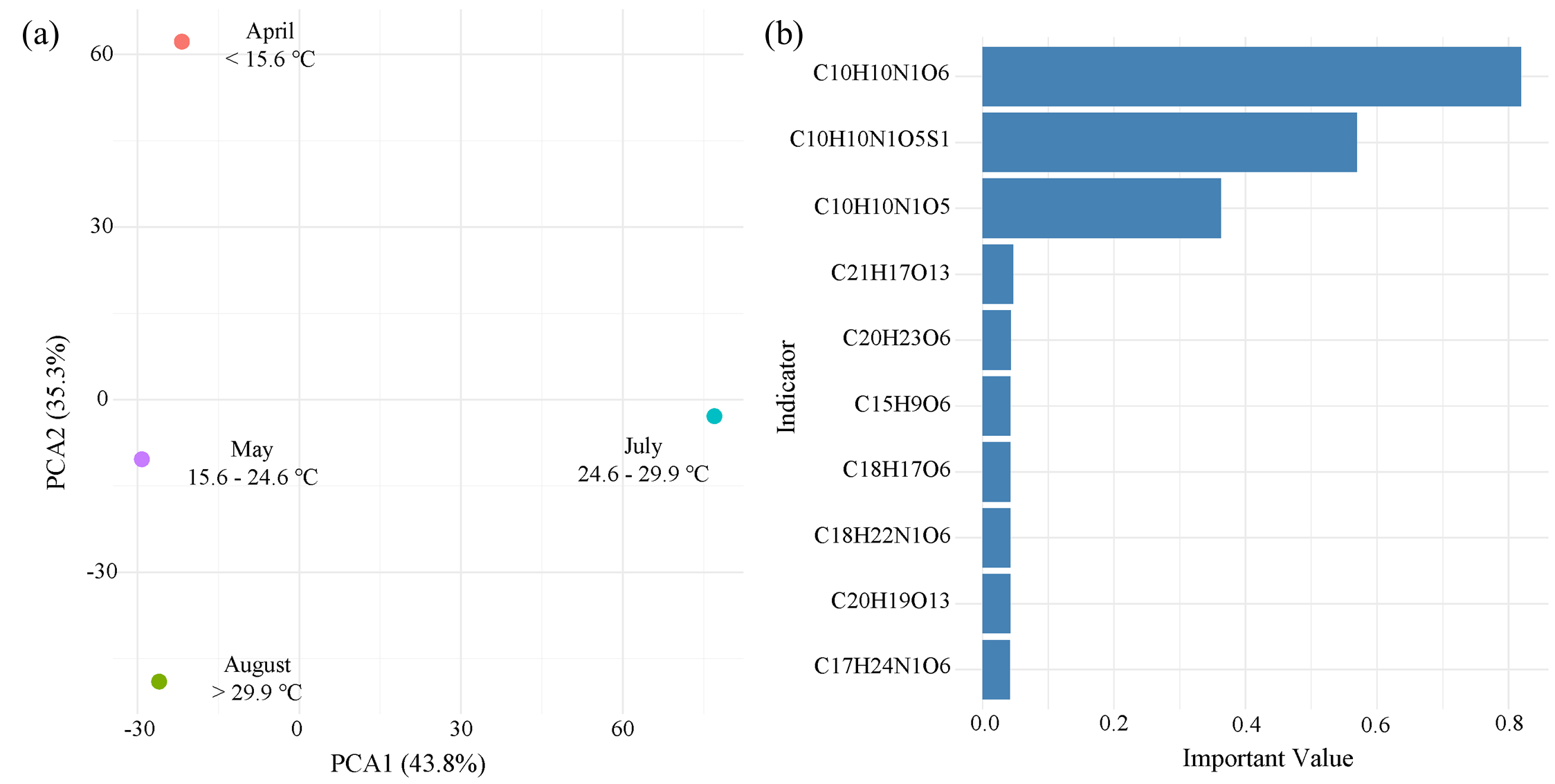


**Figure S5. Differences in DOM composition determined by FT-ICR MS among sampling months (a) and the top 10 important DOM molecules (b) based on principal component analysis (PCA).**


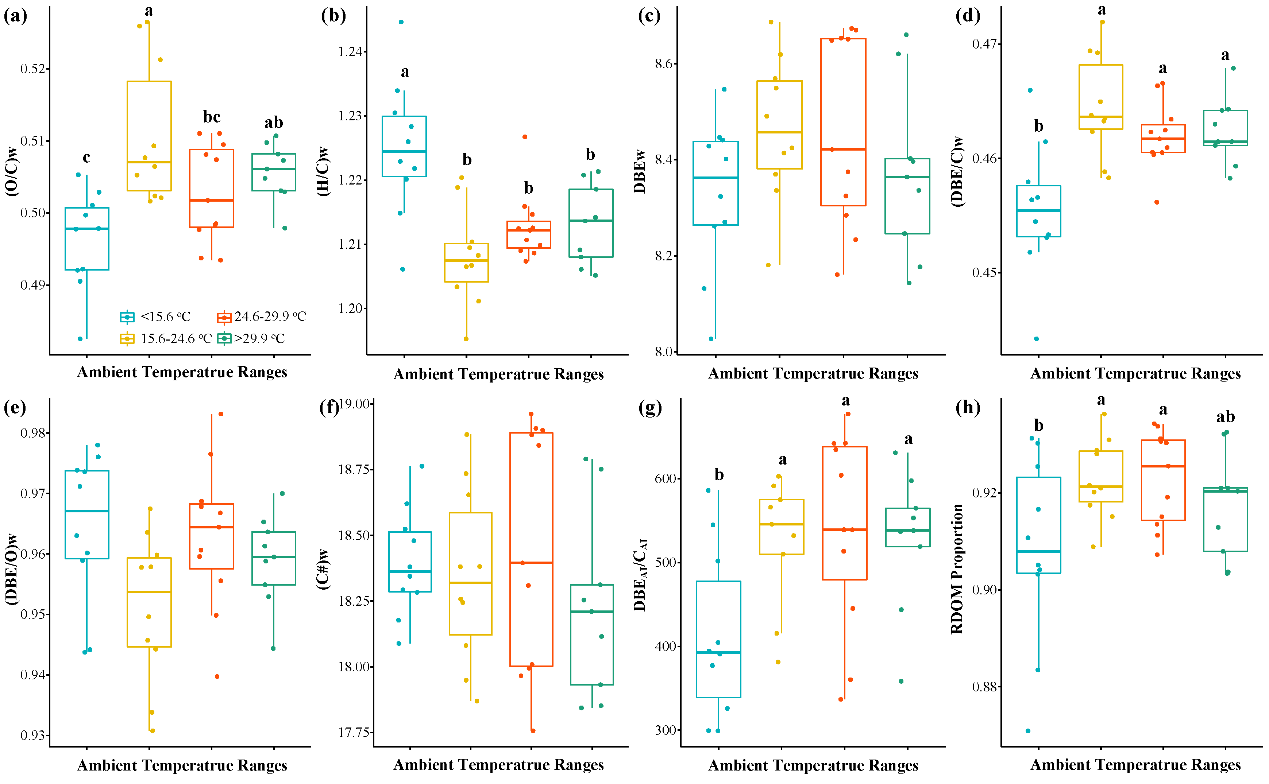


**Figure S6. Boxplots of parameters for DOM molecular formulae in different temperature ranges.** The DOM molecules represent the molecules before degradation experiments, those consumed by microbes, or those resistant to degradation. Different letters indicate a significant difference between conditions (*p* < 0.05).


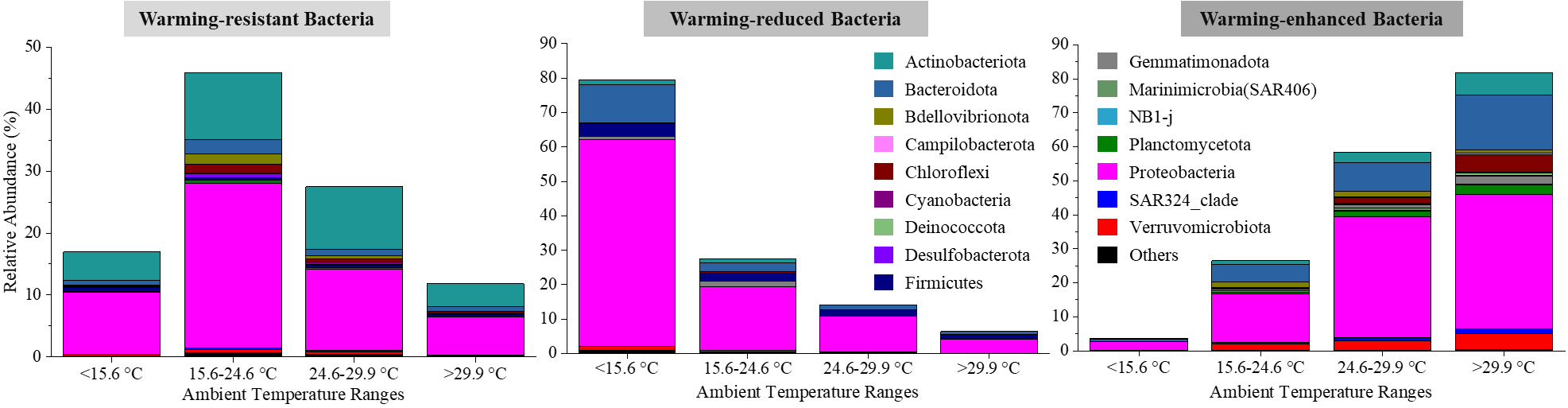


**Figure S7. Relative abundance of different warming-responsive bacteria in different temperature ranges.** Bacteria contains both attached and free-living styles. The bar chart indicates the cumulated relative abundances of the bacterial phyla in different temperature ranges.

**Figure S8. The relative abundance of bacterial phyla** **participated in the DOM transformations in different temperature ranges.** Bacteria contains both attached and free-living styles.


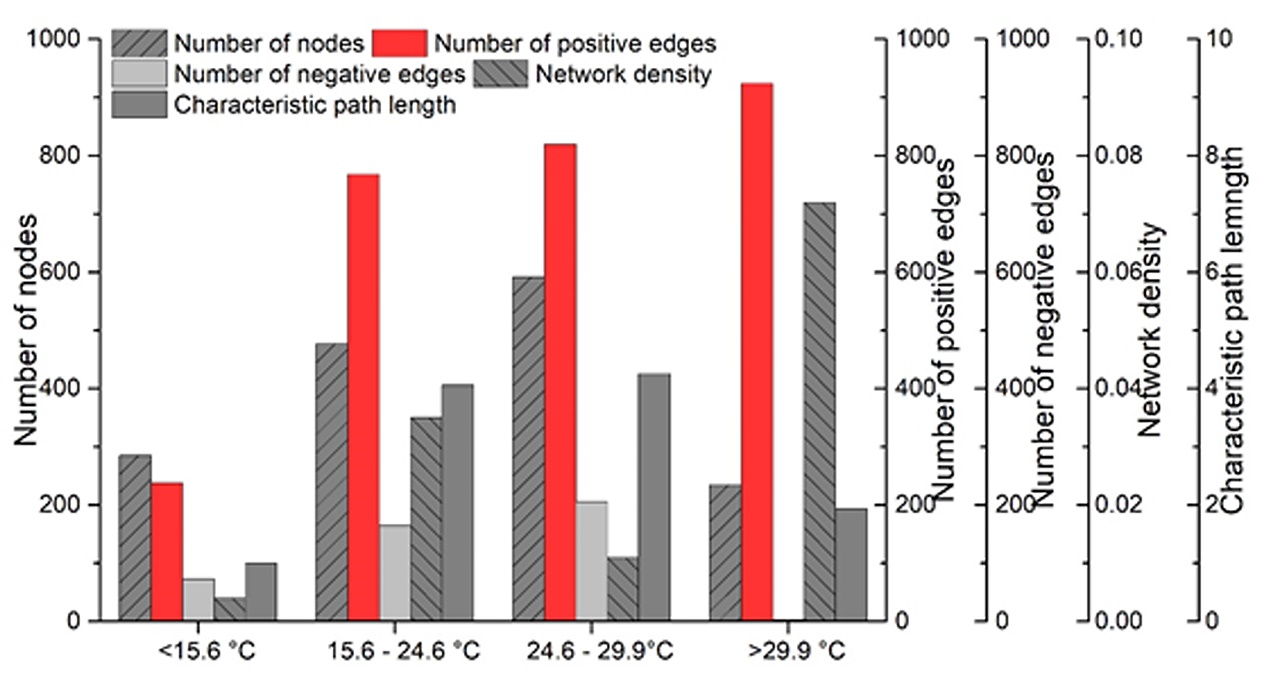


**Figure S9. Properties of co-occurrence networks for bacteria-DOM interactions in different temperature ranges.** Bacteria contains both attached and free-living styles.


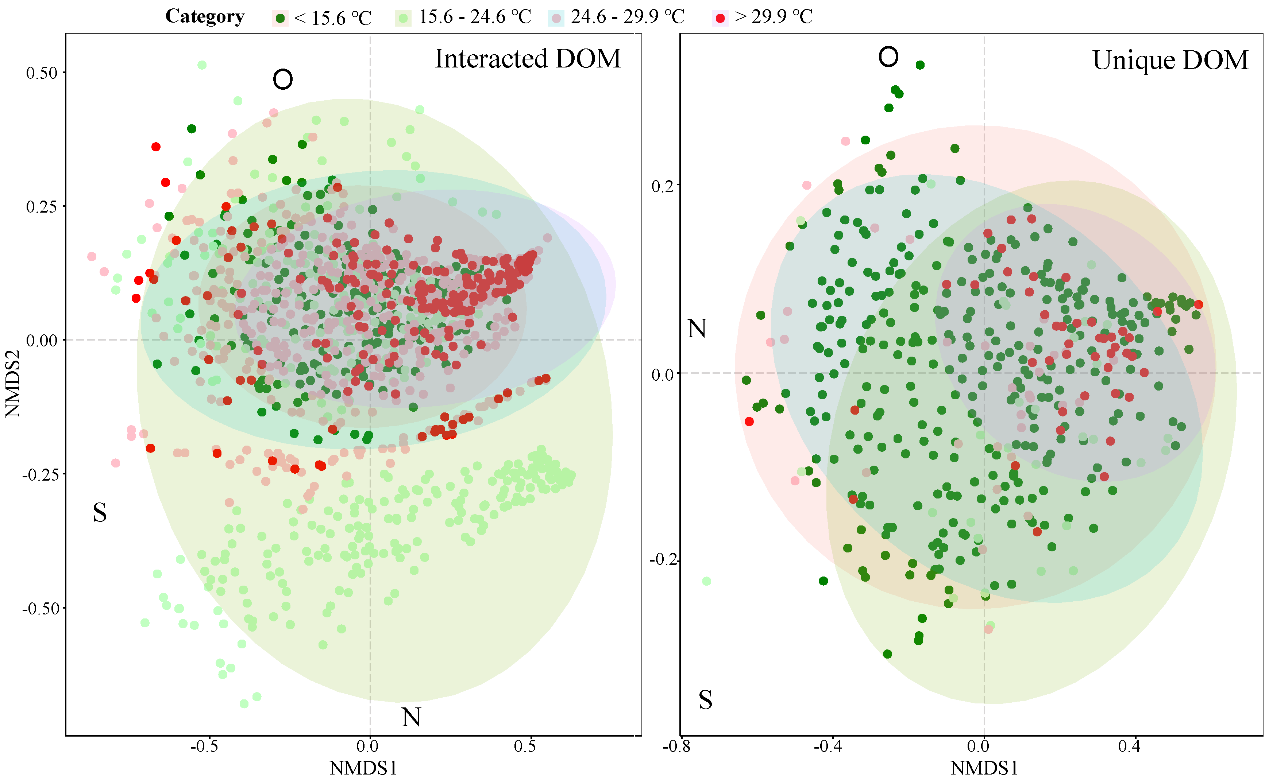


**Figure S10. Non-metric multidimensional scaling (NMDS) based on Bray-Curtis dissimilarity matrix shows the patterns of involved and unique DOM molecules.** The DOM molecules represent the molecules before degradation experiments, those consumed by microbes, or those resistant to degradation.

**Supporting Tables**

**Table S1. Parameters of fitted threshold models between the BDOC concentrations and incubation temperatures.**

| Model | RSE | R^2^ | Adjusted R^2^ | *P* |
| --- | --- | --- | --- | --- |
| Linear model (a) | 3.392 | 0.4765 | 0.4676 | <0.001 |
| One-segmented model | 3.205 | 0.5486 | 0.5248 | <0.001 |
| Three-segmented model | 2.646 | 0.7138 | 0.6761 | <0.01 |

Note: RSE, residual standard error.

**Table S2. The richness and relative abundance of DOM formulas in different temperature ranges.** The DOM molecules contain the molecules before degradation experiments, those consumed by microbes, or those resistant to degradation.

|  | < 15.6 ℃ | | 15.6 - 24.6 ℃ | | 24.6 - 29.9 ℃ | | > 29.9 ℃ | |
| --- | --- | --- | --- | --- | --- | --- | --- | --- |
|  | Richness | Relative abundance | Richness | Relative abundance | Richness | Relative abundance | Richness | Relative abundance |
| ARF | 4009 | 10.94% | 4772 | 13.01% | 5033 | 12.63% | 4379 | 12.64% |
| AAF | 0 | 0 | 0 | 0 | 0 | 0 | 0 | 0 |
| MAF | 1725 | 84.10% | 1725 | 82.23% | 1725 | 82.86% | 1725 | 82.45% |
| CRF | 4487 | 15.90% | 5250 | 17.77% | 5511 | 17.14% | 4858 | 17.55% |
| CAF | 0 | 0 | 0 | 0 | 0 | 0 | 0 | 0 |
| CRAF | 0 | 0 | 0 | 0 | 0 | 0 | 0 | 0 |

Note: ARF, always rare formulas; AAF, always abundant formulas; MAF, moderately abundant formulas; CRF, conditionally rare formulas; CAF, conditionally abundant formulas; CRAF, conditionally rare and abundant formulas.

**Table S3.** **Fractions of community assembly processes for free-living (FL) and particle-attached (PA) bacteria in different temperature ranges.**

|  |  | < 15.6 ℃ | 15.6 - 24.6 ℃ | 24.6 - 29.9 ℃ | > 29.9 ℃ |
| --- | --- | --- | --- | --- | --- |
| FL | Variable selection | 83.67% | 94.46% | 96.10% | 84.76% |
|  | Homogeneous selection | 0 | 0 | 0 | 0 |
|  | Dispersal limitation | 14.31% | 4.00% | 1.73% | 11.43% |
|  | Homogenizing dispersal | 0.60% | 0 | 0 | 0 |
|  | Undominated | 1.41% | 1.54% | 2.16% | 3.81% |
| PA | Variable selection | 45.01% | 62.46% | 47.62% | 60.95% |
|  | Homogeneous selection | 3.13% | 3.08% | 3.90% | 0 |
|  | Dispersal limitation | 44.16% | 32.31% | 45.45% | 29.52% |
|  | Homogenizing dispersal | 1.71% | 0 | 0 | 0 |
|  | Undominated | 5.98% | 2.15% | 3.03% | 9.52% |

**Table S4. Parameters of co-occurrence networks between DOM formulas and bacteria.** The DOM molecules contain the molecules before degradation experiments, those consumed by microbes, or those resistant to degradation.

|  |  | < 15.6 ℃ | 15.6 - 24.6 ℃ | 24.6 - 29.9 ℃ | > 29.9 ℃ |
| --- | --- | --- | --- | --- | --- |
| DOM | CHO | 111 (35.69%) | 334 (35.80%) | 397 (38.69%) | 327 (35.28%) |
|  | CHNO | 112 (36.01%) | 367 (39.34%) | 417 (40.64%) | 450 (48.54%) |
|  | CHNOS | 36 (11.58%) | 65 (6.97%) | 72 (7.02%) | 81 (8.74%) |
|  | CHOS | 52 (16.72%) | 167 (17.90%) | 140 (13.65%) | 69 (7.44%) |
| Bacteria | Warming-resistant | 122 (39.23%) | 500 (53.59%) | 134 (13.06%) | 1 (0.11%) |
|  | Warming-reduced | 187 (60.13%) | 263 (28.19%) | 344 (33.53%) | 143 (15.43%) |
|  | Warming-enhanced | 2 (0.64%) | 170 (18.22%) | 548 (53.41%) | 783 (84.47%) |

**Table S5. Summary of unique and involved DOM molecules in different temperature ranges.** The DOM molecules contain the molecules before degradation experiments, those consumed by microbes, or those resistant to degradation.

|  | Unique DOM molecules | | | | | | | | | Involved DOM molecules | | | | | | | |
| --- | --- | --- | --- | --- | --- | --- | --- | --- | --- | --- | --- | --- | --- | --- | --- | --- | --- |
|  |  | C | H | N | O | S | O/C | H/C | RA (‰) | C | H | N | O | S | O/C | H/C | RA (‰) |
| <15.6 | mean | 18.184 | 20.033 | 0.997 | 9.136 | 0.392 | 0.512 | 1.091 | 0.023 | 19.780 | 23.573 | 0.763 | 9.647 | 0.274 | 0.496 | 1.194 | 0.255 |
|  | SD | 5.127 | 8.990 | 1.067 | 3.131 | 0.489 | 0.142 | 0.348 | 0.012 | 4.711 | 8.244 | 1.028 | 3.077 | 0.447 | 0.144 | 0.314 | 0.708 |
|  | max | 32.000 | 43.000 | 3.000 | 16.000 | 1.000 | 1.000 | 1.909 | 0.128 | 33.000 | 45.000 | 3.000 | 16.000 | 1.000 | 0.833 | 1.933 | 7.003 |
|  | min | 9.000 | 6.000 | 0.000 | 3.000 | 0.000 | 0.167 | 0.368 | 0.002 | 10.000 | 5.000 | 0.000 | 3.000 | 0.000 | 0.176 | 0.357 | 0.006 |
|  | median | 17.000 | 19.000 | 1.000 | 9.000 | 0.000 | 0.500 | 1.082 | 0.022 | 20.000 | 23.000 | 0.000 | 10.000 | 0.000 | 0.500 | 1.214 | 0.025 |
| 15.6-24.6 | mean | 19.967 | 23.967 | 2.100 | 7.310 | 1.000 | 0.374 | 1.192 | 0.023 | 20.400 | 24.525 | 1.624 | 9.604 | 1.000 | 0.492 | 1.196 | 0.099 |
|  | SD | 4.635 | 8.568 | 0.994 | 3.253 | 0.000 | 0.162 | 0.307 | 0.009 | 6.358 | 10.124 | 0.802 | 3.150 | 0.000 | 0.158 | 0.316 | 0.224 |
|  | max | 27.000 | 43.000 | 3.000 | 15.000 | 1.000 | 0.727 | 1.722 | 0.047 | 35.000 | 49.000 | 3.000 | 17.000 | 1.000 | 1.000 | 2.091 | 1.949 |
|  | min | 10.000 | 7.000 | 1.000 | 3.000 | 1.000 | 0.158 | 0.467 | 0.005 | 9.000 | 6.000 | 1.000 | 2.000 | 1.000 | 0.111 | 0.368 | 0.006 |
|  | median | 19.500 | 23.500 | 2.500 | 7.000 | 1.000 | 0.357 | 1.177 | 0.020 | 19.000 | 24.000 | 1.000 | 9.000 | 1.000 | 0.478 | 1.231 | 0.016 |
| 24.6-29.9 | mean | 15.677 | 20.484 | 1.563 | 9.839 | 1.000 | 0.646 | 1.279 | 0.028 | 21.215 | 24.919 | 1.637 | 8.886 | 1.000 | 0.492 | 1.162 | 0.189 |
|  | SD | 3.700 | 7.663 | 0.892 | 2.437 | 0.000 | 0.147 | 0.342 | 0.023 | 6.578 | 10.568 | 0.782 | 4.232 | 0.000 | 0.142 | 0.294 | 0.512 |
|  | max | 24.000 | 33.000 | 3.000 | 13.000 | 1.000 | 0.857 | 1.917 | 0.106 | 36.000 | 51.000 | 3.000 | 17.000 | 1.000 | 1.000 | 2.091 | 4.741 |
|  | min | 9.000 | 7.000 | 1.000 | 3.000 | 1.000 | 0.200 | 0.500 | 0.005 | 9.000 | 5.000 | 1.000 | 1.000 | 1.000 | 0.167 | 0.333 | 0.002 |
|  | median | 16.000 | 24.000 | 1.000 | 11.000 | 1.000 | 0.667 | 1.375 | 0.019 | 21.000 | 23.000 | 1.000 | 9.000 | 1.000 | 0.478 | 1.185 | 0.022 |
| >29.9 | mean | 22.333 | 26.800 | 1.323 | 10.911 | 1.000 | 0.491 | 1.194 | 0.023 | 21.081 | 25.313 | 1.852 | 9.556 | 1.000 | 0.508 | 1.178 | 0.031 |
|  | SD | 4.312 | 7.765 | 0.541 | 2.548 | 0.000 | 0.105 | 0.272 | 0.009 | 6.271 | 10.670 | 0.872 | 4.044 | 0.000 | 0.144 | 0.295 | 0.063 |
|  | max | 32.000 | 43.000 | 3.000 | 15.000 | 1.000 | 0.682 | 1.813 | 0.051 | 33.000 | 45.000 | 3.000 | 17.000 | 1.000 | 0.917 | 1.714 | 0.354 |
|  | min | 10.000 | 8.000 | 1.000 | 4.000 | 1.000 | 0.269 | 0.773 | 0.005 | 8.000 | 5.000 | 1.000 | 1.000 | 1.000 | 0.143 | 0.417 | 0.003 |
|  | median | 23.000 | 28.000 | 1.000 | 11.000 | 1.000 | 0.480 | 1.200 | 0.023 | 21.000 | 24.000 | 2.000 | 9.000 | 1.000 | 0.500 | 1.222 | 0.008 |

**References**

[1] Cai W, Du Z-L, Zhang A-P, et al. Long-term biochar addition alters the characteristics but not the chlorine reactivity of soil-derived dissolved organic matter. Water Research, 2020, 185: 116260

[2] Xu W, Gao Q, He C, et al. Using esi ft-icr ms to characterize dissolved organic matter in salt lakes with different salinity. Environmental Science & Technology, 2020, 54: 12929-12937

[3] Smith CR, Sleighter RL, Hatcher PG, et al. Molecular characterization of inhibiting biochar water-extractable substances using electrospray ionization fourier transform ion cyclotron resonance mass spectrometry. Environmental Science & Technology, 2013, 47: 13294-13302

[4] Sleighter RL, Liu Z, Xue J, et al. Multivariate statistical approaches for the characterization of dissolved organic matter analyzed by ultrahigh resolution mass spectrometry. Environmental Science & Technology, 2010, 44: 7576-7582

[5] Koch BP, Dittmar T. From mass to structure: An aromaticity index for high-resolution mass data of natural organic matter. Rapid Communications in Mass Spectrometry, 2006, 20: 926-932

[6] Xue Y, Chen H, Yang JR, et al. Distinct patterns and processes of abundant and rare eukaryotic plankton communities following a reservoir cyanobacterial bloom. The ISME Journal, 2018, 12: 2263-2277

[7] Liang Y, Xiao X, Nuccio EE, et al. Differentiation strategies of soil rare and abundant microbial taxa in response to changing climatic regimes. Environmental Microbiology, 2020, 22: 1327-1340
